# Circulating serum microRNA-345 correlates with unfavorable pathological response to preoperative chemoradiotherapy in locally advanced rectal cancer

**DOI:** 10.18632/oncotarget.11649

**Published:** 2016-08-27

**Authors:** Jing Yu, Ning Li, Xin Wang, Hua Ren, Weihu Wang, Shulian Wang, Yongwen Song, Yueping Liu, Yexiong Li, Xuantong Zhou, Aiping Luo, Zhihua Liu, Jing Jin

**Affiliations:** ^1^ Department of Radiation Oncology, National Cancer Center/Cancer Hospital, Chinese Academy of Medical Sciences and Peking Union Medical College, Beijing, 100021, People's Republic of China; ^2^ The State Key Laboratory of Molecular Oncology, National Cancer Center/Cancer Hospital, Chinese Academy of Medical Sciences and Peking Union Medical College, Beijing, 100021, People's Republic of China

**Keywords:** rectal cancer, preoperative chemoradiotherapy, serum mirna, personalized treatment

## Abstract

Preoperative chemoradiotherapy (pre-CRT) has been represented as the standard treatment for locally advanced rectal cancer (LARC), but large variations of tumor radiation response to CRT have been reported in the clinic. To explore the function of microRNAs as potential therapeutic predictors of pre-CRT pathological response in LARC, we analyzed global miRNA expression in CRT-sensitive and CRT-resistant groups before treatment. MiR-345 was significantly elevated in the CRT-resistant group. Therefore, miR-345 was selected as a candidate for further analysis. We assessed the correlation between the miRNA signatures and the chemoradiotherapeutic response in 20 randomly selected LARC tissue samples (Validation set) and 87 serum samples (Training set) by qRT-PCR. Further, we validated the results in 42 randomly selected LARC serum samples (Validation set). High miR-345 expression was significantly correlated with unfavorable pre-CRT pathological response in tissue and serum. Moreover, low miR-345 levels predicted superior 3-year local recurrence free survival (LRFS). Taken together, circulating serum miR-345 correlates with unfavorable pre-CRT response and poor locoregional control in LARC. It might be a promising biomarker to facilitate patient stratification for personalized treatment.

## INTRODUCTION

Preoperative chemoradiotherapy (pre-CRT) followed by total mesorectal excision (TME) has been recommended as the standard care for patients with locally advanced rectal cancer (LARC). Patients who undergo pre-CRT achieve higher rates of R0 resection, anal sphincter preservation and lower local recurrence rates [1, 2]. Nevertheless, patients with rectal cancer exhibit heterogeneous responses to pre-CRT. Only certain subsets of patients achieving pathological down-staging or pathological complete regression (pCR) would have less risk of local recurrence and better disease-free survival (DFS) [3–6]. Given this consideration, it would be advantageous to obtain the information on the chemoradiation response before treatments initiation, which tends to stratify LARC into different response categories, and eventually optimizes a response-based therapeutic option. Therefore, it remains of utmost clinical importance to analyze the underlying mechanisms of resistance to CRT.

MicroRNAs (miRNAs) are small, highly conserved, non-coding sequences 18–25 nucleotides in length and that act as post-transcriptional regulators in tumorigenesis and development [7]. MiRNAs have important regulatory roles in cell growth, proliferation, differentiation, and death. MiRNAs are involved in different stages of colorectal cancer (CRC) pathogenesis by regulating the expression of oncogenes and tumor suppressor genes [8, 9]. Several miRNAs, including miR-21, miR-320, and miR-765 are involved in regulating CRT resistance [10–13]. Circulating miRNAs exhibit significant stability against environmental variation and could emerge as noninvasive biomarkers for diagnosing and monitoring the therapeutic effect of many diseases [14, 15]. However, few reports to date have investigated the association between circulating miRNA signatures and pre-CRT pathological response in rectal carcinoma.

To address this issue, we performed a global miRNA analysis in CRT-sensitive and CRT-resistant patients. MiR-345 was significantly elevated in CRT-resistance patients. As a potential candidate biomarker, subsequently, we investigated and validated whether miR-345 in serum correlated with unfavorable pathological response and poor prognosis in LARC before CRT initiation.

## RESULTS

### Patient characteristics

A total of 149 LARC patients were enrolled in this study. All patients were treated with concurrent CRT with doses of 50-50.4 Gy. Most patients were male (70%) and moderate differentiation (48%). The clinical stages were relatively balanced (44% stage II and 56% stage III). The clinical characteristics of the tissue validation set, serum training set, and serum validation set were summarized in Table 1. Overall, there was no significant difference among the patients in the three cohorts in terms of sex, tumor location, differentiation, pre-treatment clinical stages, or pre-CRT pathological response.

**Table 1:** Patient characteristics

Characteristics	Validation set (tissue)	Training set (serum)	Validation set (serum)	*P*
Patients	20	87	42	
Sex				
Male	11	66	28	0.149^§^
Female	9	21	14	
Median age, y (range)	51 (31-79)	55 (29-79)	54(27-80)	
Clinical stage				
II	5	42	18	0.166^§^
III	15	45	24	
Tumor location				
Low	13	50	23	0.746^§^
Middle	7	37	19	
Tumor differentiation				
Poor	5	33	10	0.364*
Moderate	9	39	24	
Good	6	15	8	
Neo-CRT response				
TRG 1/2	10	43	26	0.395^§^
TRG 3/4	10	44	16	

Abbreviations: Neo-CRT, neoadjuvant concurrent chemoradiation; TRG, tumor regression grade;

* χ^2^ test with continuity correction;

§ χ^2^ test.

### Identification of chemoradiation-related miRNA signatures

Pathological response was evaluated according to Mandard criteria [16]. TRG 1 showed absence of residual cancer and fibrosis extending through the different layers of the rectal wall; TRG 2 was characterized by the presence of rare residual cancer cells scattered through the fibrosis; TRG 3 was characterized by an increase in the number of residual cancer cells, but fibrosis still predominated; TRG 4 showed residual cancer outgrowing fibrosis.

**Figure 1:**
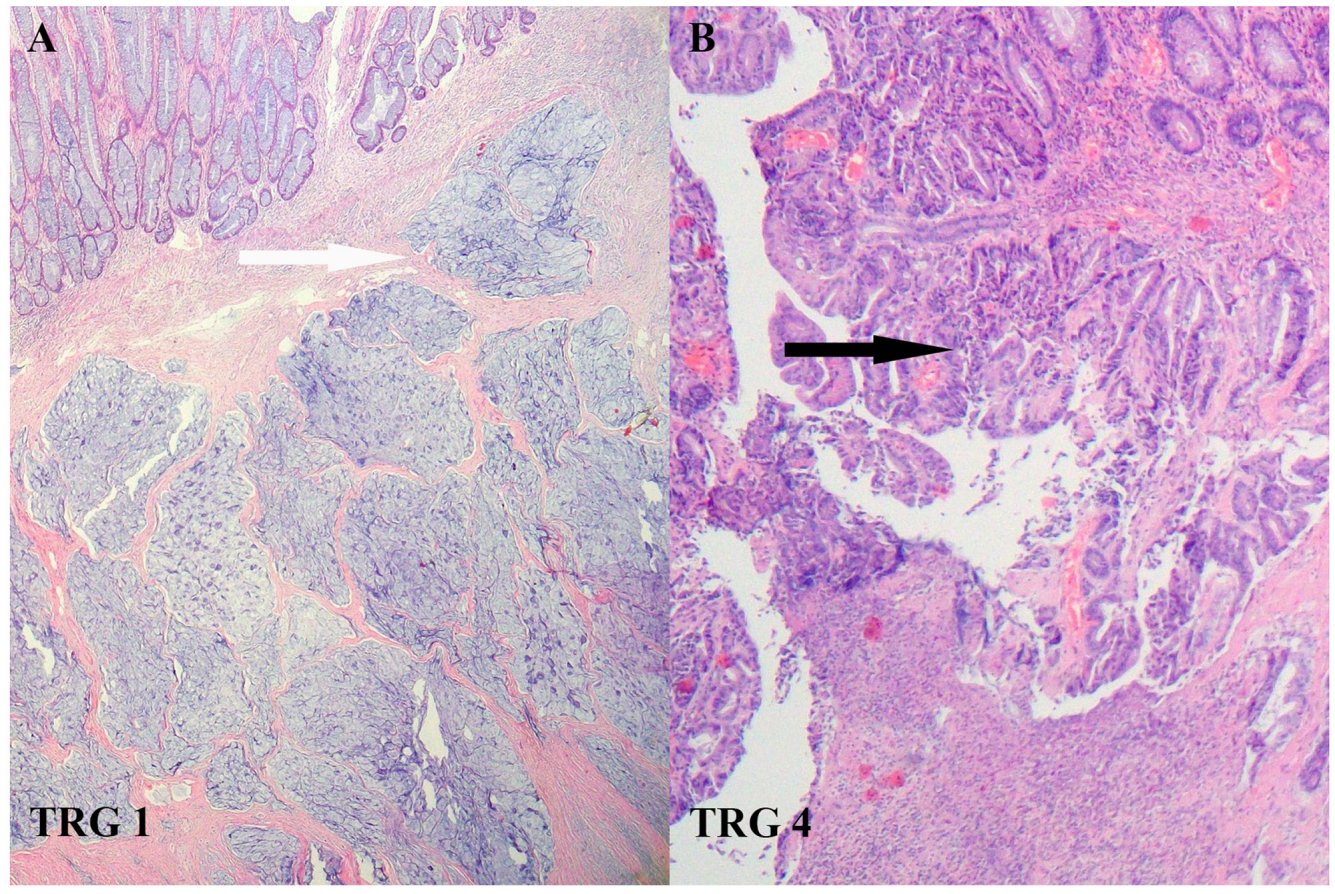
The Mandard criteria for assessing pathological response to chemoradiotherapy in LARC **A.** TRG 1 was defined as complete regression. Fibrosis had replaced large parts of the tumor (white arrow). **B.** TRG 4 indicated residual tumor outgrowing fibrosis with slight regression (black arrow).

**Figure 2:**
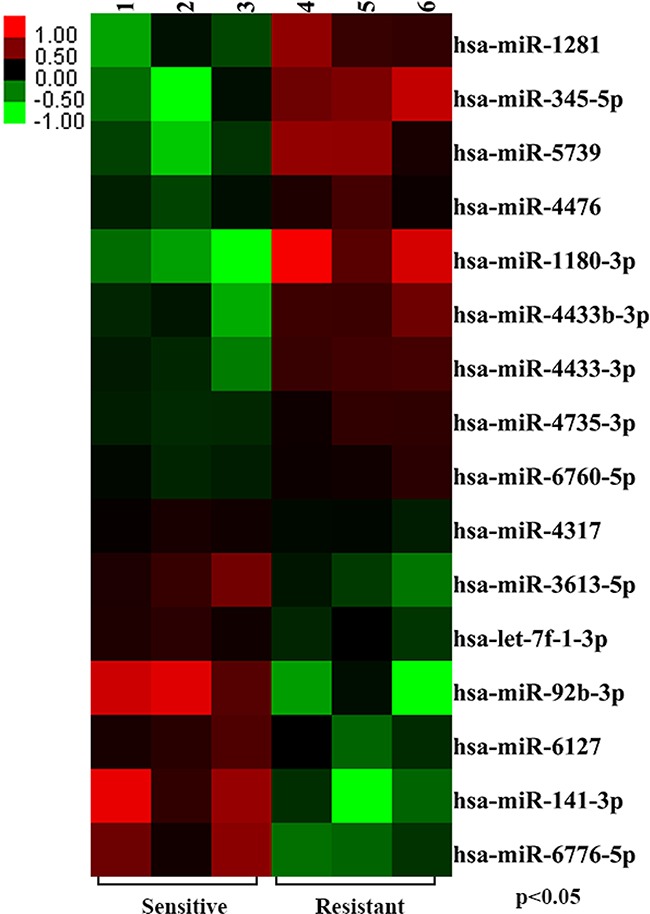
Identification of chemoradiation-related miRNA signatures The divergent miRNA expression profiles based on histopathological response were examined using miRNA array analysis in CRT-sensitive group (three samples) and CRT-resistant group (three samples). The result showed that 16 miRNAs were differentially expressed between two groups (*P*<0.05).

To determine whether miRNA expression profiles differ between CRT sensitive and resistant in LARC, we performed miRNA array between CRT-sensitive group (TRG 1) and CRT-resistant group (TRG 4) (Figure 1). All samples were pathologically reassessed by two pathologists, and had at least 70% tumor cell content. 16 miRNAs were differentially expressed between the two groups (*P*<0.05) (Table 2). Of these, miR-345-5p, miR-1180-3p, miR-1281, miR-4433b-3p and miR-5739 were consistently down-regulated. Conversely miR-92b-3p, miR-141-3p and miR-6776-5p were consistently up-regulated in the CRT-sensitive group (fold change≥2) (Figure 2). Notably, the elevation of miR-345 was the most remarkable one in patients with CRT resistance. Therefore, miR-345 was selected as a candidate for further analysis.

**Table 2:** Fold change of identified miRNAs from microarray

miRNA	Fold Change	*p-value*
hsa-miR-92b-3p	2.5479	0.019
hsa-miR-141-3p	2.1903	0.042
hsa-miR-6776-5p	1.6173	0.044
hsa-miR-3613-5p	1.4312	0.031
hsa-miR-6127	1.2973	0.044
hsa-let-7f-1-3p	1.1763	0.041
hsa-miR-4317	1.0889	0.021
hsa-miR-6760-5p	0.876	0.024
hsa-miR-4735-3p	0.8197	0.0199
hsa-miR-4476	0.8135	0.019
hsa-miR-4433-3p	0.7098	0.042
hsa-miR-4433b-3p	0.6564	0.044
hsa-miR-1281	0.6347	0.031
hsa-miR-5739	0.5636	0.044
hsa-miR-345-5p	0.4601	0.041
hsa-miR-1180-3p	0.3694	0.021

### MiR-345 as single miRNA marker for CRT resistance

Given the low incidence of TRG 1 in the entire cohort, in all three tissue validation sets, the serum training and validation sets, the radiosensitive group was defined as TRG 1/2 and the radioresistant group was defined as TRG 3/4.

**Figure 3:**
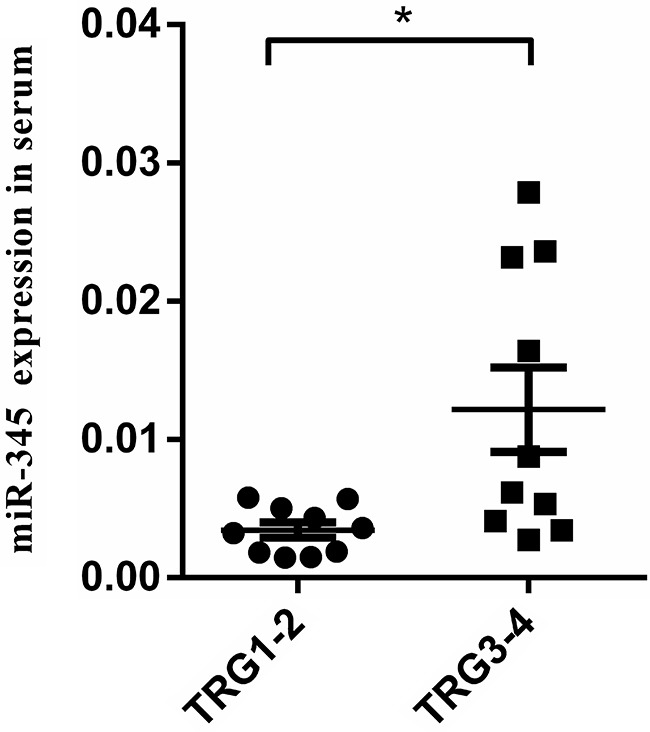
MiR-345 expression was associated with chemoradiation sensitivity in LARC tissue samples MiR-345 expression was examined by qRT-PCR in 20 LARC tissue specimens from CRT-sensitive group (TRG 1/2) and CRT-resistant group (TRG 3/4). Low miR-345 expression was associated with chemoradiation sensitivity in LARC (*P*=0.046).

To validate miRNA array data, we examined miR-345 expression using qRT-PCR in 20 randomly selected LARC tissues (10 samples were TRG 1/2, 10 samples were TRG 3/4). MiR-345 expression was consistent with miRNA array data. As shown in Figure 3, miR-345 expression was significantly down-regulated in the CRT-sensitive group compared with the CRT-resistant group (*P=*0.046).

**Figure 4:**
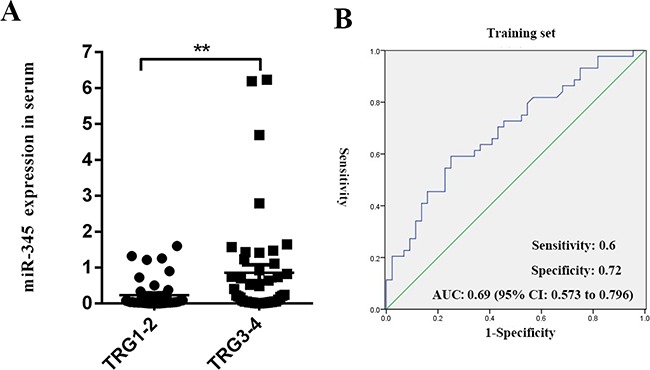
Circulating serum miR-345 expression was associated with chemoradiation sensitivity in LARC **A.** MiR-345 expression was examined by qRT-PCR in 87 LARC serum samples from the CRT-sensitive group (TRG 1/2) and CRT-resistant group (TRG 3/4) in the training set. The low expression of circulating serum miR-345 was associated with chemoradiation sensitivity in LARC (*P*=0.002). **B.** ROC curve analysis. The ROC plots for miR-345 were used to differentiate TRG 1/2 from TRG 3/4 in serum; AUC=0.69 (95% CI: 0.573-0.796); sensitivity was 60%, and specificity was 72%.

Schou et al. showed that miR-345 in whole blood could serve as a potential biomarker for clinical outcome in CRC. MiR-345 was a single prognostic biomarker for both overall survival and progression-free survival in all patients as well as the non-*KRAS* mutant population in CRC [17]. Accordingly, we focused on the potential predictive value of circulating serum miR-345. In the serum training set, low miR-345 expression was significantly correlated with CRT good response (*P*=0.002). Receiver operating characteristic (ROC) analysis of miR-345 expression yielded an area under the curve (AUC) value of 0.69 (95% confidence interval [95% CI]: 0.573-0.796, *P*<0.001) to distinguish CRT-sensitivity from resistance (Figure 4).

**Figure 5:**
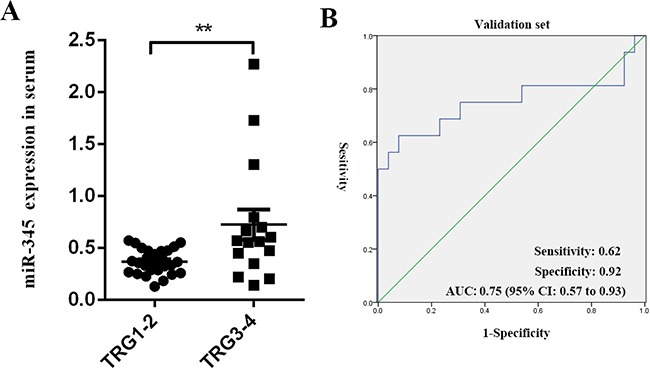
MiR-345 expression was validated in serum training set results **A.** Comparison of miR-345 expression levels in 42 serum samples (validation set) between the CRT-sensitive group (TRG 1/2) and CRT-resistant group (TRG 3/4). **P=0.007 (Mann-Whitney U test). **B.** ROC curve analysis. AUC=0.75 (95% CI: 0.57-0.93); sensitivity was 62% and specificity was 92%.

In order to validate the serum training set results, we used the same qRT-PCR method in a group of 42 randomly selected LARC serum samples (serum validation set). As expected, miR-345 expression was significantly down-regulated in the CRT-sensitive group as compared to the CRT-resistant group (*P=*0.007). ROC analysis of miR-345 expression yielded an AUC value=0.75 (95% CI: 0.57-0.93, *P*<0.01) (Figure 5). These results indicated that miR-345 in tissue or serum might act as single biomarker for CRT resistance in LARC.

### Association of serum miR-345 expression with survival in LARC

**Figure 6:**
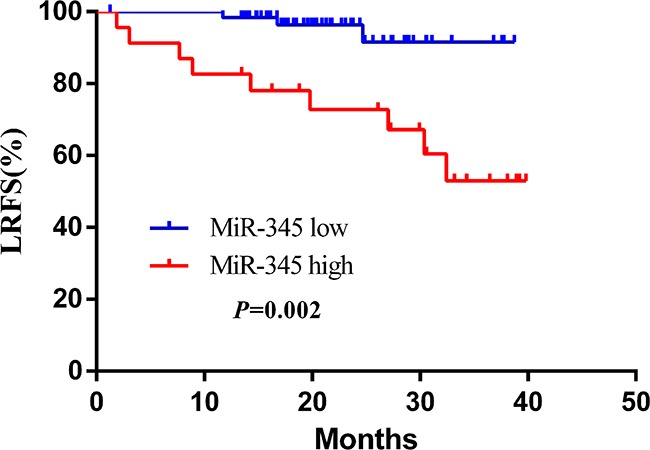
Kaplan–Meier curves of LRFS with different miR-345 expression Patients with low miR-345 expression (64 cases) had significantly higher survival rates than those with high miR-345 expression (23 cases, HR 0.14, 95% CI 0.04-0.49; *P*=0.002).

To further evaluate whether serum miR-345 levels can serve as a predictor of patient outcome, we performed Kaplan-Meier survival analysis. As shown in Figure 6, low miR-345 expression (below the mean) was correlated with superior 3-year LRFS (hazard ratio [HR] 0.14, 95% CI 0.04-0.49; *P*=0.002), but was not associated with 3-year DFS. This result indicated that circulating serum miR-345 correlates with unfavorable pathological response to pre-CRT in LARC.

## DISCUSSION

In this study, we preformed miRNA array to screen chemoradiosensity-related miRNAs from LARC tissue specimens. First, we found that the circulating serum miR-345 predicted the pathological response to pre-CRT. The low miR-345 expression in serum appeared to correlate with favorable local control in LARC. Serum miRNA signature could be obtained before initiating CRT, thus miR-345was probably used as a noninvasive predictive biomarker for prescribing a personalized treatment strategy in LARC.

Previous studies have revealed that patients with LARC who underwent pathological down-staging or pCR after pre-CRT achieved more favorable outcomes [18–21]. Regarding radiosensitivity, tumor regression or down-staging would prevent patients from having to undergo radical TME surgery, and allow them turn to the transanal local excision or “wait and watch” approaches, which could ensure therapeutic effect without unnecessary injury and could substantially improve their quality of life [20, 21]. On the contrary, radioresistant would attempt to receive dose-intensive chemotherapy regimens or higher irradiation doses to improve the therapeutic results. Thus, accurate prediction of pre-CRT pathological response before treatment has pivotal implications for the formulation of an overall therapeutic plan.

To accurately select radiosensitive patients, conventional examinations such as magnetic resonance imaging (MRI) or positron emission tomography/computed tomography were used to differentiate good radiotherapy responders from the poor responders [22–24]. However, their predictive results were likely hampered by interpretation difficulties in terms of assessing signal intensity and the presence of residual tumor within areas of radiation-induced fibrosis or edema. In addition, some particular tumor characteristics, such as total mucinous aspect, was known to exhibit hyper-intense signals on T2-weighted and diffusion-weighted MR images, which would lead to bias and bad reproducibility of prediction [25–27].

MiRNAs function as negative regulators of their target genes [28, 29]. Current studies indicate that miRNAs are modulators not strictly confined to the intracellular compartment but that are also secreted into the peripheral blood in exosomes, and regulate the expression of target genes [29]. A retrospective study showed that circulating miRNAs are highly stable and hard to be degraded by RNase. These properties facilitate their role as promising biomarkers in new cancer diagnosis, efficacy surveillance, and prognostic evaluation [30–32].

MiR-345 was first reported to be highly expressed in malignant mesothelioma tissues [33]. Subsequently, it was proven to function as the malignant transformation-related gene in oral cancer, and was probably associated with cisplatin resistance in breast adenocarcinoma [34, 35]. Moreover, miR-345 was detected in serum and predicted the adverse pathological result in prostate cancer [36]. We have identified for the first time that low expression of serum miR-345 likely contributed to the radiosensitivity to pre-CRT and lower LRFS in LARC. This observation implied that genetic factors could play an important role in influencing individual radiation susceptibility.

Recent studies have showed that serum miR-345 served as a strong adverse prognostic factor in metastatic CRC after adjusting for sex, age, KRAS, PI3KCA and performance status. Patients with high miR-345 expression were 1.75 and 1.63 times more likely to develop mortality and progression risk, and high expression was associated with poor response to chemotherapy and targeted therapy (cetuximab combined with irinotecan) [17], which was similar to our findings. Tang et al. demonstrated that low miR-345 levels were strongly correlated with poor pathological differentiation although they played an antineoplastic role in CRC [37]; that result would be an underlying explanation for our finding that LARC with low miR-345 expression is prone to radiation sensitivity. As for the low incidence of local recurrence in the serum validation set, we failed to observe the added benefit to LRFS in the cohort.

Based on our microarray analysis, miR-345 expression was significantly elevated in the CRT-resistant group, and had been identified as a circulating biomarker in our pre-experiment and by other researchers [17]. As our panel had focused on exploring the noninvasive and convenient methods for predicting the pre-CRT response for optimizing the therapeutic strategy, we only assessed the predictive value of miR-345 in LARC, which is inconsistent with previous methods.

Two recent studies confirmed that post-CRT diffusion-weighted (DW) MRI volumetry and volume reduction (Δ volume) after pre-CRT provided high and accurate diagnostic performance in assessing the good radiation response of pCR [38, 39]. We would attempt to combine the above two confounding factors (post-CRT DW imaging volumetry, Δ volume) with miR-345 expression to further improve the diagnostic performance of pre-CRT.

Previous studies showed that P21, BCL2-associated athanogene 3 (BAG3) and BCL2 were confirmed to be the targets of miR-345. Shiu et al. indicated that miR-345 could directly down-regulate the crucial tumor suppressor P21 to facilitate the hepatocarcinogenesis under the chronic HCV infection [40]. BAG3, an anti-apoptosis protein, was found to be another target of miR-345 in CRC cell lines [37]. Moreover, miR-345 was significantly down-regulated in pancreatic cancer tissues and cell lines. BCL2 was characterized as a novel target of miR-345 and its forced-expression abrogated the apoptosis effects of miR-345 in PC cells [41]. Along with the miRNA microarray analysis, we also examined the mRNA expression between CRT-sensitive and resistant groups. Combining with the correlation analysis from 6 Database, we found that 6 potential target genes including CACNA1C, RAB27B, COL17A1, TRIM58, SMAD5 and FRAT1 were to be deserve further investigation (Table 3).

**Table 3:** Potential target genes of miR-345

Source. GeneSymbol	Target. GeneSymbol	Correlation	P.value	DIANAmT	miRanda	miRDB	miRWalk	PICTAR5	Targetscan
miR-345	CACNA1C	−0.99	0.000	1	1	0	1	0	1
miR-345	RAB27B	−0.97	0.001	1	1	1	1	1	1
miR-345	COL17A1	−0.94	0.005	0	1	0	0	1	0
miR-345	TRIM58	−0.93	0.007	0	1	0	0	1	1
miR-345	SAMD5	−0.92	0.008	1	0	0	1	1	1
miR-345	FRAT1	−0.92	0.009	0	1	0	0	1	0

## MATERIALS AND METHODS

### Clinical specimens and study design

This study included 149 patients with previously untreated and histologically confirmed rectal adenocarcinoma from 2006 to 2015 at the Chinese Academy of Medical Sciences Cancer Hospital. All patients underwent standard pre-CRT plus TME. Surgery was scheduled 6–8 weeks after pre-CRT, and adjuvant chemotherapy was administered according to postoperative pathology diagnosis. This study was designed as an initial screening phase and a subsequent validation phase. For screening, we characterized the miRNA expression profiles of three neoadjuvant CRT-sensitive and three neoadjuvant CRT-resistant LARC samples using miRNA array. Pathological response was evaluated according to TRG as described by Mandard [16]. Considering the low incidence of TRG 1 in the entire cohort, we defined TRG 1/2 as CRT-sensitive and TRG 3/4 as CRT-resistant in subsequent validation sets. To identify correlation between the candidate miRNA profile and the clinical outcome, miRNA signature was first evaluated in 20 tissue samples (Validation set) and 87 serum samples (Training set) by qRT-PCR. Then, the predictive value of the candidate circulating miRNA was further validated using 42 randomly selected serum samples (Validation set).

### Ethics statement

This study protocol has been reviewed and approved by the Chinese Academy of Medical Sciences Cancer Hospital ethics committee. All participants provided written informed consent.

### MiRNA expression analysis

The pre-therapeutic biopsies from patients with LARC were stored in liquid nitrogen, and then subjected to miRNA array analysis at CapitalBio company (Beijing, China) using Affymetrix GeneChip miRNA 4.0 Arrays containing 761 miRNAs.

### Real Time Quantitative PCR

Tissue and serum samples were collected from patients before pre-CRT. Tissue RNA was isolated using TRIzol® reagent (Invitrogen, Carlsbad, CA). MiRNA extraction from 200 μl serum was performed with miRNeasy RNA isolation Kits according to the manufacturer's instructions (Qiagen, Valencia, CA). For qRT-PCR, total 1 μg RNA was reverse-transcribed with Bulge-Loop miRNA-specific reverse transcription primers using a miScript II RT Kit (Qiagen, Valencia, CA). The miR-345 looped RT primer sequence was 5′-CTCAACTGGTGTCGTGGAGTCGGCAATTCAGTTGAGGAGCCCTG-3′; the miR-345 PCR primer sequence was 5′-ACACTCCATCTGGGGCTGACTCCTAGTCCA-3′. The qRT-PCR was performed using a miScript SYBR Green PCR kit (Qiagen, Valencia, CA). Synthetic spiked-in *Caenorhabditis elegans* miR-39 was added to the serum prior to RNA extraction as the internal control. U6 small nuclear RNA was used as the internal control for the tissue samples. All reactions were run in triplicate, and miRNA expression was quantified using the comparative threshold cycle (2^−ΔCt^) method [42].

### Statistical analysis

Statistical analysis was performed using SPSS version 19.0 (SPSS Inc., Chicago, IL). Normal distribution of data was verified using the Kolmogorov-Smirnov test. The Mann-Whitney U test was used to analyze the different miRNA expression between the CRT-sensitive and CRT-resistant groups in the tissue validation set, serum training set and validation set. ROC curves were generated to evaluate the diagnostic performance in differentiating the CRT-sensitive from CRT-resistant samples. DFS was measured by the date of initial treatment to the date of disease recurrence. LRFS was evaluated from the date of surgery until the date of local or regional lymph node recurrence (or last follow-up). Survival was calculated using the Kaplan-Meier method, and compared using the log-rank test. P<0.05 was considered significant.
